# Annotations

**Published:** 1891-11-21

**Authors:** 


### Annotations.

**Think of it.**

Men of good sense and honest purpose have a great objection to sensationalism; but if there is anything they hate more than sensationalism it is cold-blooded stupidity. All the papers have told the story of the last twenty-four hours of Edward Haynes' life. Haynes was an out-of-work cooper. He appears to have done all he could to find work. The last effort he made was to walk from Tunbridge to London, a distance of something over thirty miles. The journey would carry him past hundreds of rich men's houses and grounds. The whole country, in fact, between Tunbridge and the outskirts of London is dotted with such houses. What poor Haynes must have thought as he passed carriages rolling along the road, drawn by well-fed horses, with their occupants clad in the warm clothing and rich furs of the season; what he must have thought as he looked at the clean maid-servants with their trim caps and other marks of happy comfort, we may all imagine. He, himself, was homeless, he had no friends, no money, and no food. Doubtless, he was too proud to beg, and his pride, a not unworthy pride, proved to be his death. He struggled up to London, and avoiding the parts where the rich and prosperous dwell, betook himself to the haunts of the poor. He must have known that a casual ward at a poor house would receive him if he made application. He would not make application. He preferred to walk the streets, although he was starving with hunger and cold. Apparently, by some good chance, he found a refuge for the night from the cold and rain. The refuge consisted but of four walls and a roof. It had neither food to give to the wayfarer nor a bed for him to lie upon. He who sought its shelter must go supperless to rest on a bare floor or a wooden chair, with all his rain-soaked rags clinging close about him. One hundred and ninety-three wayfarers like Haynes had sought this melancholy hospice of despairing poverty on the night when he was its guest. Haynes was passed on from the shelter to the workhouse door; then to bed, to warmth, comfort, and good nursing for a few hours; then to death, to the Coroner's court, and to a pauper's grave. "Willing to work; too proud to beg; died of starvation; in a Christian land." Such might be the epitaph of poor Edward Haynes. He has passed beyond the reach of suffering; like Lazarus, we trust, to Abraham's bosom. But what of us fairly prosperous people who are still left here, with votes and a civilised state, in charge of one another and of our weaker brethren who have dropped out of the ranks of regular work? Might not even a little sensationalism be permitted and encouraged if it would force us to think of the workless poor, and to take steps for providing them with work, or with food and shelter until work could be found? The Poor Law has proved itself to be insufficient: it is not the right thing. In another connection a great Christian writer once said: "We are not under the law, but under grace." There is a wonderful depth of meaning in these words. Law, bare, hard law, was found inadequate for the development of the higher manhood of the world. Grace, love, had to be called in. Law, too, is manifestly unfit to cope with the rugged pride and noble independence of the better part of our English working classes. We must devise some means of dealing with them in a spirit of brotherliness. The thing must be done. Our churches and manifold religious organisations stand disgraced until it is done. Christ never uttered a profounder truth or a truth more necessary for the higher development of man and the conservation of noble races than when he said: "Inasmuch as ye have not done it unto one of the least of these my brethren, ye have not done it unto Me." We have not done it in the past, or we have but done it partially. No; but we must do it now; and we must do it thoroughly and efficiently, as we so well know how to do it.

**Genius and Insanity.**

Professor Cesare Lombroso, of the University of Turin, has written a book which he calls "The Man of Genius." The book has given rise to a good deal of adverse criticism. It is not, however, the first absurd book that has ever been published, nor is it likely to be the last. But we are accustomed to expect from University Professors, if not brilliancy and wit, at least science and sense. Professor Lombroso disappoints these not unreasonable expectations. Of course, it is open to a University Professor to publish arrant nonsense if he so pleases; but it is a pity that a University should be compelled to stand sponsor for offspring which is hardly worthy of the poorest Mechanics' Institute or Hall of Science of the slums. "Genius," says the Professor, "has been classed by not a few alienists as on the confines of criminality, one of the teratologic forms of the human mind, a variety of insanity." This is pure stupidity. It looks like wilful and ostentatious silliness, as if a man should say to himself—"I will be a fool, and I will make the world think me an extremely clever and scientific fellow." What, after all, is genius? It is simply a superior capacity for doing one or many things. But the things have usually to be done by means of the brain as an instrument; and it is a fact, perfectly well established, that the more work and the higher work that is done by any particular brain, the more likely is that brain to exhaust itself, and to become in time incapable of any work at all; to become, in fact, used up, unsound, insane. It is not the natural brain of the man of genius that is in a condition of insanity, it is the exhausted and used-up brain. The athlete uses up his heart and his muscles—and why? Because, feeling conscious of extraordinary power of heart and muscle, he takes undue liberties with these endowments. Similarly, the man of genius, feeling conscious of extraordinary brain power, is tempted to work at full gallop, and often does work, like poor Scott from necessity, or like Stuart Mill from choice, until he has exhausted and worn out his splendid thinking instrument. Professor Lombroso puts Scott and Mill, with Gibbon, Erasmus, Linnæus, John Hunter, Wilberforce, Browning, George Eliot, and many others into a common limbo of mental unsoundness. For what reason? Chiefly because they were one and all abnormally little of stature. This is science, if you please; and University science. One thing is pretty certain, that, whether Professor Lombroso succeeds or not in convincing us of the insanity of Wilberforce, Hunter, and Stuart Mill, he will certainly succeed in convincing most sober readers of his own ridiculousness. So far from genius being an evidence of insanity, it is a proof of superior soundness of mind. If the man of genius would but use his brain as the man of sense uses his high-mettled hunter, and work it with moderation, he need never fear insanity—except, of course, of the hereditary kind, which all men of insane families may fear. The great lesson that brain-workers who have become insane teach us is—not that genius and insanity are twins, or even parent and child, but that unusual brain power requires to be used with discretion, and that excessive mental activity should be balanced and counteracted by abundant brain rest and adequate physical exertion. Professor Lombroso has convincingly demonstrated to the world that science and stupidity make a very ill-matched pair of legs.

**A Venerable Volume.**

The important medical manuscript which Georg Ebers acquired at Luxor eighteen or nineteen years ago, only fragments of which has hitherto been translated, is now, we learn, accessible to all who can read German. A complete translation has been made by a Berlin medical man, Dr. Henrich Joachim, who learned Egyptian for the purpose. By his assiduous labours the oldest medical work in the world, written, it is supposed, at latest 1550 B.C., and many parts of which are of a much older date, is now common property. It consists mainly of receipts, interspersed here and there with proverbs. The treatise shows that many methods at present in use were practised by the old Egyptian physicians.

